# Caesarean section rates in Mozambique

**DOI:** 10.1186/s12884-015-0686-x

**Published:** 2015-10-12

**Authors:** Qian Long, Taina Kempas, Tavares Madede, Reija Klemetti, Elina Hemminki

**Affiliations:** Global Health Research Centre, Duke Kunshan University, No. 8 Duke Avenue, 215316 Kunshan City, Jiangsu Province P.R.China; Family Federation of Finland, PO Box 849, FI-00101, Helsinki, Finland; Department of Community Health, Eduardo Mondlane University, 702 Salvador Allende Ave, C.P 257, Maputo, Mozambique; National Institute for Health and Welfare, PO Box 30, FI-00271, Helsinki, Finland

**Keywords:** Caesarean section rate, Underuse, Overuse, Health system research, Mozambique

## Abstract

**Background:**

The Caesarean section (C-section) rate is used as an indicator for availability and utilization of life-saving obstetric services. The purpose of the present study was to explore changes in C-section rates between 1995 and 2011 by area, place of delivery and maternal socioeconomic factors in Mozambique.

**Methods:**

Cross-sectional data from the Demographic and Health Surveys conducted in Mozambique in 1997, 2003 and 2011 were used, including women having a live birth within 3 years prior to the survey. Descriptive statistics and logistic regressions were used to identify factors associated with having a C-section.

**Results:**

The C-section rate decreased slightly from 2.5 % in 1995–1997 to 2.1 % in 2001–2003 and then increased to 4.7 % in 2009–2011. In 2009–2011, C-section rates ranged in urban areas from 4.6 % in the northern region to 12.2 % in the southern region and in rural areas from 1.6 % in the northern region to 3.9 % in the southern region. 12.3 % of the richest women had had a C-section, compared to 1.7 % of the poorest women. C-sections were the most common at public hospitals (12.6 % in 2009–2011), but C-sections at health centers increased from the second to the third period. The likelihood of having a C-section was associated with living in urban areas and in the southern region, having a formal education and living in a rich household, even adjusting for age and parity (and study periods). The strongest relationship was for the richest household wealth quintile [OR (95 % CI): 9.8 (6.3–15.3)]. The highest rate (20.6 %) was found among the richest women giving birth at public hospitals in the southern region in 2009–2011.

**Conclusion:**

In Mozambique, underuse of C-section was likely among the poor and in rural areas, but overuse in the most advantaged groups seemed to be emerging.

**Electronic supplementary material:**

The online version of this article (doi:10.1186/s12884-015-0686-x) contains supplementary material, which is available to authorized users.

## Introduction

Essential and emergency obstetric care has been prioritized politically in many low- and middle-income countries in order to attain the Millennium Development Goals (MDGs) of reducing maternal and child mortality and morbidity [1]. The number of Caesarean sections (C-sections) as a percentage of all births is used as an indicator for measuring availability and utilization of life-saving obstetric services [2]. The World Health Organization (WHO) suggests that the C-section rate should not be less than 5 % or more than 15 %, although no optimum rate has been determined [2, 3]. The WHO further noted that the upper level is not a target to be achieved but a threshold to be controlled [2]. Both very low and very high C-section rates are associated with adverse maternal and neonatal health outcomes [4–6].

In the past decades, the C-section rate has increased rapidly in many developing countries, mostly in Latin America and some in Asia, largely due to non-medical factors such as physicians’ interest in profits, schedule planning, defensive medicine and possible maternal wishes [4, 7–10]. In the 1990s, the C-section rate ranged from 17–40 % in 12 Latin American countries where most births occurred at health care facilities [11]. In Peru, C-section rates increased at all health facilities between 1991 and 2008, but the greatest increase was observed in the private sector: from 28 % in 1991–1999to 53 % in 1999–2008 [10]. In Asia, China has the highest C-section rate (46 %), followed by Vietnam (36 %) and Thailand (34 %) [4]. C-sections have increased notably in some countries in southern Asia (e.g. Bangladesh, India and Nepal) in recent years [12]. It has been argued that proliferation of private health services might contribute to increase of C-sections, together with a high facility-based C-section rate in these countries [12].

In most African countries, concern has focused on the underuse of C-sections. A recent study examined changes in the C-section rate in sub-Saharan Africa over time, using data from the repeated Demographic Health Surveys (DHS) [12]. The study reported that C-section rates had increased overall in the vast majority of African countries, but C-section rates in most of those countries were still lower than 5 % even in 2007–2011. Socio-economic inequality in access to C-section was remarkable [7, 12, 13]. We have found no studies on C-section rate by place of delivery and across region within a country in Sub-Saharan Africa. These would be very important for measuring progress in improving access to C-section from the perspective of equity in health care.

Mozambique is a low-income country in southern Africa. In 2009, half of the population lived below the national poverty line [14]. In recent years, there has been strong economic growth [15]. The maternal mortality ratio decreased from an estimated 910 deaths per 100,000 live births in 1990 to 490 in 2010 [16]. In Mozambique, the public sector is the main healthcare provider. There have been substantial efforts to provide comprehensive obstetric care integrated in the national health services system, supported by a number of international donors [17, 18]. The main facilities in the public sector are health centers and hospitals (secondary and higher levels). The staffing and functions of health centers depend on the area, but usually they are staffed with unspecialized physician and nurses. Most health centers provide birth services, but not usually C-sections or other major surgery. Larger health centers have inpatient beds. Obstetric specialists are few [17, 18], but since 1984 Mozambique has trained assistant medical officers to perform major obstetric surgery, especially in rural areas [19]. In the public health sector, C-section is free of charge to women if they comply with the referral regulation. In recent years, private hospitals have gradually emerged in big cities, but the role of the private sector in maternity care is not well known.

There are three level nurses providing essential obstetric care. Usually high level and middle level maternal and child health nurses (MCH nurses) have 5 years and 2.5 years MCH training after high-school, respectively. Basic nurses have 1.5 yeas MCH training after high-school. Over the years schooling system has changed and health facilities have various types of health personnel.

In Mozambique, the DHS surveys were conducted in 1997, 2003 and 2011, and birth data from the 2011 survey are not reported in other studies. The present study used the DHS data generated from the three survey periods to explore changes in C-section rates by area, place of delivery and maternal socioeconomic factors in Mozambique between 1995 and 2011. The present study adds new evidence of variation in C-section coverage within the country and contributes to the international debate on health policy development for improving maternal and child health in sub-Saharan African countries.

## Methods

Demographic and Health Surveys were conducted in Mozambique by the National Statistics Institute and the Ministry of Health of Mozambique in 1997, 2003 and 2011; technical support was provided by the Measure DHS project team [20–22]. We obtained permission from the team of Measure DHS. Birth datasets of the Mozambique surveys were downloaded from the website of the Measure DHS (https://dhsprogram.com/data/dataset_admin/login_main.cfm?logout=&CFID=31936610&CFTOKEN=78613481). The purpose of the DHS is to collect nationally representative data on population, health and nutrition for policy formation, program planning, monitoring and evaluation. The DHS system is funded by the U.S. Agency for International Development (USAID) and has been conducted in over 90 countries.

The three surveys employed the same multi-stage, stratified sampling procedure. The enumeration areas for the country defined in the cartography of the Mozambique Population Census were used as the primary sampling units, usually comprising 100–200 households each. The probability proportional sampling method was used to randomly select primary units: 975 in 1997, 604 in 2003 and 611 in 2011. In urban units 20 households and in rural units 25 households were randomly selected. In each household, all women aged 15–49 years and men aged 15–64 years were eligible for the survey, as defined by the DHS team.

The interviewers were hired and trained by the National Institute of Statistics; women were interviewed at home. In each year, women’s response rates were over 90 %, and the number of interviewed women was 8,779 in 1997, 12,414 in 2003 and 13,718 in 2011. Women having a live birth within 3 years prior to the survey – 3,255 in 1997, 5,414 in 2003 and 6,256 in 2011 – were included in the study. If a woman had more than one delivery within the study period, the last one with at least one live birth was included.

The questionnaire was divided into several sections, including information on the general demographic and socioeconomic background, fertility, family planning, reproductive and sexual health, child health and HIV/AIDS. In each survey, there was a section headed ‘Pregnancy and Breastfeeding/Postnatal Care’, including information on the birth history and utilization of maternity care in the last delivery. The questions in the three surveys were similar.

The outcome measure was whether the birth occurred by a C-section. In 1997, C-section was surveyed with the question “Was the [name] (baby) born by an operation of cutting your belly? 1) yes; 2) no”. In 2003, the question was “Did the birth [of name] take place by normal (vaginal) delivery, or operative vaginal delivery with assisted suction cup, or caesarean? 1) normal delivery; 2) operative vaginal delivery with suction cup; 3) caesarean section”; in 2011, it was: “Was [name] delivered by caesarean, that is, did you undergo a surgery to take the baby out? 1) yes; 2) no”. In the three surveys, the DHS team had generated a binary variable of having or not having a C-section in the datasets.

The explanatory variables were: type of residence (urban or rural), geographic region (North, Central and South), place of delivery (home, public health center or post (hereinafter collectively referred to as health centers; “births at health post” only reported in the 1997 survey), public hospital, other (including private health facility and other non-specified facility), maternal age, education (no education, primary education and secondary or higher education, readily grouped by the DHS team), parity (total number of children ever born) and in the 2003 and 2007 surveys household wealth index (quintiles). For the geographic region, the 11 provinces were grouped into North (Niassa, Cabo Delgado and Nampula), Central (Zambezia, Tete, Manica and Sofala) and South regions (Inhambane, Gaza, Maputo province, Maputo city). In 2009, the Gross Domestic Product (GDP) per capita in the South region was two times that of the Central region and almost 2.5 times that of the North region. The wealth index based on household assets (including durable consumer goods, quality of the home, and water and sanitation facilities) was calculated by the DHS team using principal components-derived weights [23]. Households were classified into five wealth quintiles (poorest, poor, middle, richer, richest), each containing one fifth of the households interviewed.

We calculated population-based C-section rates in total and in various subgroups as the number of women giving birth by C-section divided by the number of women having live births. For a sub-analysis, we calculated the facility-based C-section rate: the number of women giving birth by C-section at a certain type of health facility divided by the total number of women giving birth at that type of facility. C-section rates were compared between the study periods, urban and rural areas, regions and by maternal demographic and socio-economic characteristics and place of delivery.

The DHS team calculated sampling weights based on sample design parameters to correcting non-response or other calibrations. We made analysis both using the sampling weights and not using the sampling weights, and found very similar results. There were also inconsistences over the three surveys in some variables (e.g. rural and urban areas) in the DHS datasets. Considering the sampling weights are specific to a single wave of the survey, we decided to present data without the use of sampling weights. The chi-square test was used to check the statistical significance of proportions. Logistic regressions were used to analyze the association between having C-section and explanatory variables, firstly adjusting for maternal age and parity and secondly additionally adjusting for the study year.

## Results

### Background characteristics and place of delivery

Table 1 presents the numbers and characteristics of women who gave birth in the three years prior to each survey. Most of the women lived in rural areas; the percentage of urban women increased between the first and the second study period, but did not increase further by the third study period. The distribution of women between the three geographical areas was relatively stable, the share of women living in the central region modestly increasing. The distribution of maternal age and parity was relatively similar in the three study periods. The percentage of women without formal education decreased from the second to the third study period; in the third study period, 16 % of women had received secondary or higher education. The proportion of twin or multiple births increased from the second to the third study period, being 4 % in the third study period.

**Table 1:** Background characteristics of women in the study, women having given birth within three years prior to the DHS surveys in 1997, 2003 and 2011, Mozambique, % of women

	1997 (n = 3255)	2003 (n = 5414)	2011 (n = 6256)	*P value1, 1* ^*st*^ *vs 2* ^*nd*^ *period*	*P value2, 2* ^*nd*^ *vs 3* ^*rd*^ * period*
	%	%	%		
Type of residence					
Urban	23.4	35.3	31.6	*<0.01*	*<0.01*
Rural	76.6	64.7	68.4		
Age					
14–19	19.1	19.2	18.5	*0.44*	*0.23*
20–25	33.9	32.5	31.9		
26–29	18.5	18.3	17.8		
30+	28.5	30.0	31.8		
Education					
No education	39.0	40.6	33.5	*<0.01*	<0.01
Primary	57.3	53.6	50.9		
Secondary +	3.7	5.8	15.6		
Parity					
1	23.4	21.7	21.7	*0.12*	*0.66*
2–3	35.7	35.0	34.8		
4+	40.9	43.3	43.5		
Twin or multiple births					
Yes	1.6	2.2	4.0	*0.11*	*<0.01*
No	98.4	97.8	96.0		
Place of delivery^a^					
Home	50.3	43.2	35.9	*<0.01*	*<0.01*
Public health center	25.1	36.0	38.0		
Public hospital	24.0	18.8	22.8		
Other	0.7	2.0	3.3		
Living region					
North	27.3	27.0	26.1	*0.17*	*<0.01*
Central	38.9	40.8	43.8		
South	33.8	32.2	30.1		

^a^Place of delivery: “public health center”: 7 % of births occurred at a public health post in the 1997 survey, but none in the 2003 survey, and there was no “public health post” category in the 2011 survey; “other” includes private and non-specified health facilities

Information on the “place of delivery” was missing for 11 women in 1997, 5 women in 2003, and 86 women in 2011

The percentage of women giving birth at home decreased from 50 % in the first study period to 36 % in the third period, more births occurring at public health centers (Table 1). The percentage of women giving birth at public hospitals did not increase. Births occurring at other health facilities increased over time, but their percentage remained small. “Other facilities” is a heterogeneous group, its most common members being non-specified facilities. The group also contains private hospitals; in 2003 and 2011, 0.3 % and 0.2 % of births occurred in them, respectively.

Home births were much rarer in urban than rural areas, but similar decreasing trends were found in both areas. In urban areas, between the first and the second study periods, the share of women who had given birth at public hospitals decreased with more births occurring at public health centers (Additional file 1). In rural areas, even in the third study period, around half of women gave birth at home. Births occurring at public health centers increased over time. In all geographical regions, home births decreased and births occurring at public health centers increased over time (Additional file 1). Home births were least common and births at public hospitals most common in the southern region.

There was a strong correlation between birth place and household wealth: in the two surveys with this data, over 90 % of women from the richest households gave birth at health facilities and half of them gave birth at public hospitals, while 37 % of women from the poorest household gave birth at health facilities and only 6 % of births occurred at public hospitals (Additional file 1).

### C-section rates

As a mean, C-section rates were low, but there were major differences by area and the women’s characteristics. The rates declined slightly from the first (2.5 %) to the second period (2.1 %), and then modestly increased to 4.7 % in the third period (Table 2). In all three periods, women in urban areas were more likely to have a C-section than women in rural areas. Women in the southern region had higher C-section rates than women in the central and northern regions. In the last period, C-section rates ranged in urban areas from 4.6 % in the northern region to 12.2 % in the southern region and in rural areas from 1.6 % in the northern region to 3.9 % in the southern region (Table 2).

**Table 2:** C-section rates by region, urban/rural area and place of delivery, women having given birth within three years prior to the DHS surveys in 1997, 2003 and 2011, Mozambique (%)

Region		1997 (*n* = 3224)^a^	2003 (*n* = 5411)^a^	2011 (*n* = 6256)^a^	*P value1 1* ^*st*^ *vs 2* ^*nd*^ *period*	*P value 2 2* ^*nd*^ *vs 3* ^*rd*^ *period*
Nationwide	Overall	2.5	2.1	4.7	*0.19*	<0.01
	Urban	6.7	4.7	9.6	*<0.05*	<0.01
	Rural	1.2	0.6	2.5	*<0.05*	<0.01
	*P value 3*	<0.01	<0.01	<0.01		
North	Overall	1.7	1.3	2.3	*0.42*	<0.05
	Urban	9.3	3.5	4.6	*<0.05*	0.42
	Rural	0.8	0.4	1.6	*0.28*	<0.01
	*P value 3*	<0.01	<0.01	<0.01		
Central	Overall	1.7	0.6	3.7	*<0.01*	<0.01
	Urban	5.0	1.6	8.7	*<0.01*	<0.01
	Rural	1.0	0.2	2.4	*<0.01*	<0.01
	*P value 3*	<0.01	<0.01	<0.01		
South	Overall	4.0	4.5	8.3	*0.51*	<0.01
	Urban	6.9	7.5	12.2	*0.73*	<0.01
	Rural	2.1	1.6	3.9	*0.46*	<0.01
	*P value 3*	<0.01	<0.01	<0.01		
Place of delivery	Home	0.6	0	0	*--*	--
	Public health center	2.5	0.4	4.3	*<0.01*	<0.01
	Public hospital	6.6	9.8	12.6	*<0.05*	<0.05
	Other	0	3.7	9.3	*0.36*	0.07

^a^Information on “whether a woman had or did not have C-section” was missing for 31 women in the 1997 survey and 3 women in 2003

C-section rates at public health centers decreased from the first to the second period and then increased in the third period; C-section rates at public hospitals and other health facilities increased over time (Table 2). A similar trend was observed by urban and rural area and by region. The highest C-section rate at public hospitals was 17 % in the southern region (Additional file 1). The percentage of C-sections at private hospitals was 22 % in both the second and third period, but the numbers of births was small (18 in the second and 9 in the third period).

The overall trend was observed in most socio-economic sub-groups (Table 3). Women who had secondary or higher education and were wealthy were more likely to undergo a C-section than other women (Table 3). There were no statistically significant differences in C-section rates by age. Women having their first child were more likely to have a C-section.

**Table 3:** C-section rate by women’s characteristics, women having given birth within three years prior to the DHS surveys in 1997, 2003 and 2011, Mozambique (%)

	1997 (n = 3224)^a^	2003 (n = 5411) ^a^	2011 (n = 6256) ^a^	*P value 11* ^*st*^ *vs 2* ^*nd*^ *period*	*P value 2 2* ^*nd*^ *vs 3* ^*rd*^ *period*
Age					
14–19	1.9	3.0	5.6	*0.20*	<0.01
20–25	2.9	1.7	4.4	*<0.05*	<0.01
26–29	3.5	1.7	4.7	*<0.05*	<0.01
30+	1.6	2.1	4.6	*0.41*	<0.01
* P value 3*	0.07	0.09	0.46		
Education					
No education	1.4	0.8	2.2	*0.10*	<0.01
Primary	3.1	2.3	4.2	*0.12*	<0.01
Secondary +	5.1	8.3	11.8	*0.26*	0.09
* P value 3*	<0.01	<0.01	<0.01		
Parity					
1	3.6	3.8	7.8	*0.78*	<0.01
2–3	2.2	2.0	4.2	*0.76*	<0.01
4+	2.1	1.2	3.6	*<0.05*	<0.01
* P value 3*	0.09	<0.01	<0.01		
Wealth quintile					
Poorest	--	0.2	1.7	--	*<0.01*
Poor	--	0.3	2.3	--	*<0.01*
Middle	--	0.7	2.7	--	*<0.01*
Richer	--	2.1	4.1	--	*<0.01*
Richest	--	7.2	12.3	--	*<0.01*
* P value 3*	--	<0.01	<0.01		

^a^Information on “whether a woman had or did not have C-section” was missing for 31 women in the 1997 survey and 3 women in 2003

Household wealth was a strong predictor for C-section, and the socio-economic disparity was notable. In the last period, 12 % of the women from the richest households had a C-section, as opposed to only 2 % of the women from the poorest households. The C-section rate among the richest urban women was 13.7 % in the southern region, compared to 1.3 % among the poorest rural women in the northern region. The C-section rate at public hospitals ranged from 18.6 % among the richest women to 5.5 % among the poor women (Additional file 1). Among the most advantaged women who were the richest women in the southern region and gave birth at public hospitals, the C-section rate was 20.6 %.

Adjusting for both maternal age and parity did not influence the results relative to place of residence, region, education, wealth or place of delivery as reported above (Table 4). But when adjusting for parity, older women were found to be more likely to give birth by C-section than younger women. Furthermore, the difference in regard to parity became greater after adjusting for maternal age.

**Table 4:** Likelihood of having C-section by women’s background characteristics, women having given birth within three years prior to surveys in 1997, 2003 and 2011 Mozambique, crude likelihood and adjusted results by logistic regression, odds ratio (OR) and 95 % confidence interval (CI)

	Crude OR (95 % CI)	Adjusted OR^a^ (95 % CI)	Adjusted OR^b^ (95 % CI)
Years			
1995–1997	1.00	1.00	--
2001–2003	0.82 (0.62–1.10)	0.84 (0.63–1.13)	–
2009–2011	1.95 (1.52–2.51)	1.98 (1.54–2.54)	--
Place of residence			
Rural	1.00	1.00	1.00
Urban	4.93 (4.06–5.98)	4.58 (3.77–5.57)	4.45 (3.66–5.41)
Region			
North	1.00	1.00	1.00
Central	1.21 (0.91–1.62)	1.20 (0.90–1.61)	1.19 (0.89–1.58)
South	3.41 (2.62–4.43)	3.02 (2.32–3.94)	3.08 (2.36–4.01)
Age			
14–19	1.00	1.00	1.00
20–25	0.80 (0.62–1.02)	1.28 (0.98–1.67)	1.28 (0.98–1.67)
26–29	0.87 (0.65–1.15)	2.22 (1.58–3.11)	2.22 (1.58–3.11)
30+	0.80 (0.62–1.03)	2.85 (1.98–4.10)	2.77 (1.92–3.98)
Education			
No education	1.00	1.00	1.00
Primary	2.28 (1.77–2.93)	2.31 (1.79–2.98)	2.29 (1.78–2.96)
Secondary+	7.86 (5.95–10.4)	6.60 (4.93–8.85)	5.74 (4.26–7.74)
Parity			
1	1.00	1.00	1.00
2–3	0.53 (0.43–0.66)	0.39 (0.31–0.50)	0.39 (0.31–0.50)
4+	0.44 (0.35–0.54)	0.21 (0.15–0.29)	0.21 (0.15–0.29)
Wealth quintile			
Poorest	1.00	1.00	1.00
Poor	1.52 (0.88–2.64)	1.52 (0.88–2.63)	1.42 (0.82–2.47)
Middle	1.93 (1.15–3.25)	1.95 (1.16–3.28)	1.86 (1.11–3.13)
Richer	3.52 (2.19–5.68)	3.44 (2.13–5.54)	3.25 (2.01–5.24)
Richest	11.81 (7.60–18.4)	10.34 (6.63–16.1)	9.80 (6.28–15.3)
Place of delivery ^c^			
Public health center	1.00	1.00	1.00
Public hospital	4.43 (3.59–5.47)	4.22 (3.42–5.20)	4.41 (3.57–5.45)
Other	2.86 (1.81–4.52)	2.77 (1.75–4.38)	2.53 (1.59–4.01)

^a^Adjusted for maternal age and parity

^b^Adjusted for maternal age, parity and year

^c^Place of delivery (excluding home births): “public health center”: 7 % of births occurred at a “public health post” in the 1997 survey, but none in the 2003 survey, and there was no “public health post” category in the 2011 survey; “other” includes private and non-specified health facilities

When adjusting additionally for the study year, similar results to those adjusted only for age and parity were found (Table 4). Thus, the correlates in each time period were the same.

 The same analysis as in Table 4 was performed including only facility-based delivery. The results were similar to those described in Table 4, but the odds ratios were usually somewhat smaller (data not shown).

## Discussion

In Mozambique, the Caesarean section (C-section) rate decreased slightly from 1995–1997 (the first study period) to 2001–2003 (the second study period), and then modestly increased to 2009–2011 (the third study period). C-sections were more common in the southern than the northern or central regions. Facility-based deliveries, particularly at health centers, increased remarkably during the study period. C-section rates varied by the type of facility; the decline from the first to the second study period was due to the decline in rates at health centers. In all study periods, the C-section rate was higher among urban, more educated and wealthier women. The highest C-section rate was found among the richest women giving birth at public hospitals in the relatively developed southern region (20.6 %) which is beyond the upper limit of the C-section rate recommended by the WHO (15 %).

### Strengths and limitations

In general, Demographic and Health Survey (DHS) data are considered to be highly standardized and produce good, nationally representative data. We have no information on the data quality in the three Mozambique surveys, but an assessment of birth history data in DHS surveys in 22 developing countries (not including Mozambique) in 1990 indicated reasonably accurate and complete information [24]. However, there were some limitations in the DHS data used. The question on the mode of delivery by C-section differed slightly in the three surveys. However, it is unlikely that women would forget or misreport a childbirth involving a surgical procedure. The categories of place of delivery were not exactly the same in the three surveys. For example, the alternative “health post” occurred only in the 1997 and 2003 surveys. In each survey, births occurring at non-specified health facilities were reported. In the analysis, we grouped these and births occurring at private sector facilities into the category “Other”. In the latest study period, the variable “place of delivery” was more often missing than in the first and second study periods. We do not know the reason for this. It is possible that the women could not easily identify the new types of health facilities emerging in recent years.

### Decrease in C-section rate from the first to the second period

We found that there was an overall decrease in the C-section rate from the first to the second study period. At the same time, there was a rapid increase in the percentage of births at health centers in both urban and rural areas and in all regions. According to the population census in 2007, the total number of births increased by a factor of almost 1.5 from 1995–1997 to 2001–2003 [25]. Thus, the number of births at health centers increased notably. Taking into account the increased number of births at health centers, the numbers of C-sections at health centers declined less, even though the rates declined notably. The number of births at hospitals increased much less, but the number of C-sections in the southern region increased.

In the 1990s, the Mozambique government had reformed the health system, and as a part of this process trained nurses/assistant medical officers were assigned to health centers and rural hospitals around the country to promote equitable access to primary health care including maternal health care [18, 19, 26]. However, because of the increased number of births at health centers, even the increased human resources were not enough to respond to the need, resulting in a proportional decrease in C-sections. In addition, along with the health system reform in Mozambique, the decision was also made to decentralize human resource management in the health sector, aiming to improve the speed and efficiency of processing health workforce documentation (including appointments, movement orders, pays and among others) and administrative activities [27]. However, one study reported that the decentralization of human resources management did not help but instead resulted in delaying the assignment of health professionals, mainly due to issues related to financial and administrative management [27].Taking into account the increased number of C-sections at southern hospitals, it is very likely that more highly trained professionals had found employment there. Qualified obstetricians and technicians would be likely to stay in the relative developed southern region [28], and it is also logical that the delay in the human resource management process would be shortest in the region closest to the Ministry of Health.

### Increase in the C-section rate from the second to the third period

Between the second and the third period, the overall C-section rate increased from 2.1 % in 2001–2003 to 4.7 % in 2009–2011. Apparently this was due to three factors: more women giving birth at hospitals, more surgery available and expanding private care with financial incentives to surgical delivery. With support from international organizations, the Mozambican Ministry of Health introduced target interventions, including equipping health facilities with surgical supplies and training health professionals in obstetric surgery and anesthesia [17–19]. In addition, many health professionals capable of doing C-sections (“technicians”) were imported from other countries [29, 30]. Between 2002 and 2010, per capita total expenditure on health increased from $34–$65 in Purchasing Power Parities (PPP) international dollar in Mozambique [31].

Private health services, particularly in big cities, have emerged. This includes private services integrated in public hospitals [32]. Between 2002 and 2011, private expenditure (including voluntary health insurance and direct payments for health care) as a percentage of total health expenditure increased from 30 %to 58 % [31]. In our study, the number of births in the private hospital was very small. We were not able to identify private services in public sectors. But high C-section rates at public hospitals in urban areas in the developed southern region and among the women from the richest households suggest that some of this may be due to private care within the public sector.

In addition, the C-section rate among women with their first child significantly increased from the second to the third periods. This may predict an increased need for repeat C-sections and require a response from the health care system.

### Underuse and overuse of C-section

Victora’s study (2012) in 35 ‘Countdown to 2015’ countries concluded that the spread of interventions and technologies to improve mother and child health tended to be pro-rich rather than pro-poor. As a mean, the C-section rate in Mozambique was relatively low, but there were significant differences by maternal socioeconomic characteristics. Consistent with other studies in sub-Saharan African countries and other low- and middle-income countries [7, 9, 12, 13], women who lived in rural areas, had less education and were from poor households were less likely to have a C-section. We found that C-section rates among the poorest women and women with the least education were less than or around 2 %, even in the last study period (2009–2011). Regional differences persisted over time. The C-section rates in the least developed northern region in rural areas were less than 1 % in the first and second periods and 1.6 % in the last period. It is quite likely that with these overall low rates there were many women who did not get a C-section even though it would have benefited her or her baby. Supplies and health professional constraints are still the main barriers to providing obstetric care, resulting in inaccessible or inadequate access to C-sections in many sub-Saharan African countries, especially among socially vulnerable groups [12, 33, 34].

On the other hand, relatively high C-section rates among some social groups suggest the emergence of overuse of C-section in this poor country. Physicians’ interest in profits, schedule planning, defensive medicine and possible maternal wishes have been reported as common reasons for non-medically indicated C-sections in many middle- and low-income countries [4, 7–10]. Unnecessary C-sections involve medical risks for mothers and infants [4, 5] and also take away medical resources from necessary medical care, including medically necessary C-sections for other women. In Mozambique, one fourth of public sector health professionals had a second practice in a private context to increase their income [35]. In Maputo city, dual practice among obstetricians in the city was common [Long Q, data from an unpublished study]. Dual practice may jeopardize the availability and quality of obstetric care in the public sector*.*

## Conclusion

Underuse of C-sections, particularly among socially vulnerable groups who are poor, and/or living in rural areas and poor-resource regions, is still a central concern in Mozambique. Conversely, overuse of C-sections by the most advantaged groups who are rich and living in big cities seemed to be emerging. Our results hence suggest that the provision of basic and emergency obstetric services need to be carefully designed to address current shortage of services and avoid health resources wasting on unnecessary services. To improve maternal and child health, targeted interventions are urgently needed to inform health policy development and to plan health programs. This is particularly important in the poor country with insufficient health resources.

## Additional file

Additional file 1:
**Appendix 1.** Percentages of facility-based births ^(a)^ and the C-section rate of facility-based births (%) by area, women having given birth within three years prior to the DHS surveys in 1997, 2003 and 2011, Mozambique. **Appendix 2.** Percentage of facility-based births ^(a)^ and C-section rate of facility-based births (%) by household wealth quintile, women having given birth within three years prior to the DHS surveys in 2003 and 2011, Mozambique. (PDF 122 kb).

## Abbreviations

C-section: Caesarean section
DHS: Demographic and Health Survey
MCH: Maternal and Child Health
MDGs: Millennium Development Goals
PPP: Purchasing Power Parities
USAID: U.S. Agency for International Development
WHO: World Health Organization
