# Supplementary material for: Caesarean section rates in Mozambique
Source: BMC Pregnancy Childbirth. 2015 Oct 12;15:253. doi: 10.1186/s12884-015-0686-x (PMC4603730; doi:10.1186/s12884-015-0686-x)
Supplement: Additional file 1: — Appendix 1. Percentages of facility-based births (a) and the C-section rate of facility-based births (%) by area, women having given birth within three years prior to the DHS surveys in 1997, 2003 and 2011, Mozambique. Appendix 2. Percentage of facility-based births (a) and C-section rate of facility-based births (%) by household wealth quintile, women having given birth within three years prior to the DHS surveys in 2003 and 2011, Mozambique. (PDF 122 kb). [file 12884_2015_686_MOESM1_ESM.pdf]

Appendix 1. Percentages of facility-based births [(a)] and the C-section rate of facility-based births (%) by area, women having given birth within three years prior to the DHS surveys in 1997, 2003 and 2011, Mozambique.

		1997		2003		2011		*P value ,1st vs 2nd period*	*P value , 2nd vs 3rd period*
		(n=3244)	(n=3216)[b]	(n=5409)	(n=5406)[b]	(n=6170)	(n=6170)[b]		
		Births %	C-section %	Births %	C-section %	Births %	C-section %		
Between urban and rural									
Urban	Public health center	27.4	4.4	40.5	0.4	37.0	6.5	*<0.01*	*<0.01*
	Public hospital	56.5	8.8	44.4	9.9	47.1	14.7	*0.50*	*<0.01*
	Other	0.53	0	1.8	8.8	3.1	11.7	*0.54*	*0.67*
P value 3			*0.11*		*<0.01*		*<0.01*		
Rural	Public health center	24.4	1.8	33.5	0.4	38.4	3.3	*<0.01*	*<0.01*
	Public hospital	14.0	4.1	4.8	8.9	11.5	8.5	*<0.05*	*0.88*
	Other	0.72	0	2.1	1.3	3.4	8.3	*0.62*	*<0.05*
P value 3			*0.09*		*<0.01*		*<0.01*		
Across regions									
North	Public health center	21.6	1.6	28.2	1.0	38.6	2.7	*0.52*	*<0.05*
	Public hospital	11.6	8.1	14.1	7.3	13.3	7.9	*0.81*	*0.81*
	Other	0.23	0	1.5	0	2.1	12.1	--	*0.09*
P value 3			<0.05		<0.01		<0.01		
Central	Public health centre	29.7	2.2	38.5	0.2	36.6	5.6	*<0.01*	*<0.01*
	Public hospital	16.0	5.0	11.0	4.5	18.2	8.3	*0.82*	*0.06*

	Other	0.56	0	1.99	0	3.14	7.1	--	*0.07*
P value 3			0.16		<0.01		0.12		
South	Public health center	22.8	3.6	39.3	0.3	39.4	3.8	*<0.01*	*<0.01*
	Public hospital	43.0	7.0	32.6	12.9	37.7	17.0	*<0.01*	*<0.05*
	Other	1.2	0	2.5	9.3	4.6	10.5	*0.25*	*0.84*
P value 3			0.12		<0.01		<0.01		

[a] Place of delivery: “public health center”: 7% of births occurred at a “public health post” in the 1997 survey, but none in the 2003 survey, and there was no “public health post” category in the 2011 survey; “other” includes private and non-specified health facilities. Information on “place of delivery” was missing for 11 women in the 1997 survey, 5 women in 2003 and 86 women in 2011.

For the proportions of home births, see Table 1.

[b] Information on “C-section” was missing for 39 women in the 1997 survey, 8 women in 2003 and 86 women in 2011.

Appendix 2. Percentage of facility-based births [(a)] and C-section rate of facility-based births (%) by household wealth quintile, women having given birth within three years prior to the DHS surveys in 2003 and 2011, Mozambique.

		2003		2011		*P value, 2nd vs 3rd period*
		(n=5409)	(n=5406)[b]	(n=6170)	(n=6170)[b]	
		Births %	C-section %	Births %	C-section %	
Poorest	Public health center	23.7	0.7	28.2	3.2	*<0.05*
	Public hospital	4.1	2.0	5.6	8.1	*0.16*
	Other	2.4	0	3.5	10.5	*0.07*
P value 3			0.55		<0.05	
Poor	Public health center	32.1	0.3	33.9	5.3	*<0.01*
	Public hospital	3.9	5.4	9.1	5.5	*1.00*
	Other	2.1	0	2.0	0	
P value 3			*<0.01*	0.51		
Middle	Public health center	42.5	0.4	39.5	3.5	*<0.01*
	Public hospital	7.8	7.1	14.4	6.8	*0.84*
	Other	1.8	0	5.0	8.1	*0.54*
P value 3			<0.01		0.09	
Richer	Public health center	47.3	0.4	46.7	3.6	*<0.01*
	Public hospital	25.9	7.1	29.1	7.5	*0.84*
	Other	1.8	5.3	3.0	10.0	*0.54*
P value 3			<0.01		<0.01	
Richest	Public health center	35.3	0.3	39.8	5.6	
	Public hospital	54.6	12.5	51.8	18.6	*<0.01*
	Other	2.0	14.3	3.1	15.0	*<0.01*
P value 3			<0.01		<0.01	*0.94*

[a] Place of delivery: "public health center": 7% of births occurred at a "public health post" in the 1997 survey, but none in the 2003 survey, and there was no "public health post" category in the 2011 survey; "other" includes private and non-specified health facilities. Information on "place of delivery" was missing for 5 women in the 2003 survey and 86 women in 2011.
For the proportions of home births, see Table 1.
[b] Information on "C-section" was missing 8 women in the 2003 survey and 86 women in 2011.
